# An Account of a Suppression of Stools and Urine, Occasioned by an Accumulation of Hardened Fæces in the Rectum

**Published:** 1786

**Authors:** Isaac Oliphant

**Affiliations:** Surgeon in London


VI. *An Account of a Suppreffion of Stools and Urine, occafioned by an Accumulation of hardened Fæces in the Rectum. By Mr.* Ifaac Oliphant, *Surgeon in London.*

MR. P—, the patient, whofe cafe I am about to relate, was in his fixty-fixth year, of a coftive habit, robuft, and of a fwarthy complexion, till about the latter end of November, 1784, when he became ruddy in the face. His good ftate of health, however, continued; but his wife expreffed her fears that that ſome diſeaſe was portended by this change of complexion. Accordingly, at the latter end of January following, he was attacked with an inflammation of the membrane lining the meatus externus auditorius, which ended in ſuppuration. Under both of thoſe ſtates he was in great miſery from the pain, and during the latter, was troubled with much offenſive diſcharge.

For this complaint various means were employed by different practitioners, for ſeveral months, without material benefit. At the beginning of October, the pain was extended to the muſcles moving the lower jaw, which was now in a great meaſure locked, as it were, and could not be exerciſed in the extent of motion it was capable of, without conſiderable pain; on which account he could not maſticate his uſual food, and was therefore obliged to lay aſide a ſort of coarſe wheaten bread he had hitherto made uſe of to prevent coſtiveneſs.

On the 25th of October he was directed to take a grain of opium at bed time, with a view to relieve the pain in the ear; and for ſome days this doſe was ſufficient for the purpoſe, but it ſoon became neceſſary to increaſe it to two grains; and this doſe was continued till

Novem-

November the 18th, when he was adviſed to cover the ear with a ſaturnine poultice, which afforded him great relief.

The opium at this time cauſing obſtinate conſtipation, notwithſtanding the uſe of Glauber's ſalt, of which he had generally taken half an ounce every day, and the pain, by the uſe of the poultice, being much leſſened, he almoſt entirely left off the opium.

On the 12th, on account of his coſtiveneſs, he was adviſed to take a table-ſpoonful of Caſtor oil, which ſoon paſſed through his bowels, and was voided with ſome liquid contents tinged with fæces.

During the 13th, 14th, and 15th, he was getting worſe and worſe with reſpect to ſtools, and he had only twice a little liquid diſcharge after the 12th.

On the 16th he could not void his urine freely, or in any tolerable quantity. In the morning of the following day he made only a little water; and in the courſe of this day he was ſeized with pain about the anus, which gradually increaſed in violence, with frequent attacks of teneſmus (but without any evacuation by ſtool) which were attended with a racking pain, that obliged him to cry out till the efforts ceafed.

On the 18th, the attacks recurred with more frequency and feverity, attended with pain in the back and pubis, and with a great defire to make water, as at the time he was relieved he had not paffed a drop for forty-three hours.

This day, while I was abfent, an apothecary was called in, who fent him a laxative mixture, to relieve what he fuppofed to be the inward piles, and advifed him to fit over the fteam of warm water.

In the evening being informed of his fituation, and that the fuppreffion of urine ftill remained, I took with me catheters and bougies to relieve him. I found him in one of his efforts to expel his ftool and urine, and crying out from the agony, which lafted a confiderable time after the endeavours went off. His pulfe at this time beat only forty-five ftrokes in a minute.

On afking him a few queftions, and taking an external view, I fufpected the cafe to be a collection of hardened fæces in the rectum, and that the complaint was of long ftanding; the imagined ftools that he had on the 12th and ſince, ſeeming to have been only an eſcape of the thin contents of the inteſtines between the ball of excrement and rectum, ſeveral caſes of which kind had occurred to, and uſed to be related in his lectures, by the late Dr. Hunter, whoſe memory muſt be ever gratefully revered by his pupils.

I procured ſome oil and common paper, and after beſmearing my fore-finger with the oil, it was introduced into the rectum, and there I found the impediment, a large plug of hard fæces. The finger was puſhed into the middle of this, and hooked, and every poſſible manœuvre uſed to break it down into leſs pieces, many of which were brought away, but not without great pain to the patient. When about half was extracted, and the remainder much divided, as far as could be reached, a clyſter of warm water was attempted to be thrown up, but before the whole of it was injected, we were obliged to deſiſt, from his having a motion to go to ſtool. The effort to evacuate went off, however, but returned in five minutes, when an effectual evacuation took place, and a quantity of urine flowed out insenſibly. On examination, there was found to remain a large large mafs, which was divided, and foon after voided in the fame manner as the firft.

A mixture of ol. ricini, manna, and foluble tartar, in mint water, was now prefcribed, and removed the coftivenefs effectually; and on the 19th, he was well with refpect to his bowels, and the voiding of his urine, a little forenefs only remaining.

The account of the ear-ach, and of the remedies employed to relieve it, feemed to me neceffary to give an adequate idea of all the circumftances of the cafe. The exiftence of hardened fæces, is perhaps not fufficiently attended to; and I have heard of one inftance in which it was overlooked by feveral practitioners, but was at laft fuccefsfully removed. Cafes of this kind, however, do not always terminate fo happily; and I remember the late Dr. Hunter ufed to relate the cafe of a lady advanced in years, in whom fymptoms of irritation came on, the caufe of which was fufpected to be a fchirrus in the pelvis; and the real nature of the complaint was not afcertained till after death, when it was found to have depended on indurated fæces.

The obftruction of the urine in the cafe I have related was certainly mechanical, from the fæcal ball preffing on the membranous part of the urethra; for, as foon as part of the ball was voided, the urine was infenfibly poured forth, and was afterwards freely dif-charged by the regular action of the bladder, when the impediment was entirely removed.

The florid countenance fupervening at this patient's period of life, feems to give an idea of the manner of an attack of apoplexy, which would moft likely have been the cafe here, (as he had many of the characterizing marks of predif-pofition to this difeafe,) if the bulk of blood had been as much increafed in the internal circula-tion of the head, as it was in the external; for, in the former it would, perhaps, have induced hemorrhage; whereas in the latter it probably difpofed only to inflammation.

The above cafe may ferve as a leffon to us to inveftigate complaints about the parts con-cerned in it, by manual certainty. There furely is no difagreeable fubmiffion a practi-tioner ought to evade that has a real tendency to relieve his afflicted patients. I am fatisfied it was the only alternative in the above cafe; and in fimilar circumftances the finger appears to be the beft inftrument we can employ; for independent of the confined room from the contraction contraction of the ſphincter ani, the rectum in this caſe was ſo irritable, that even the cautious inſinuation of the finger produced great pain, and this muſt have been much increaſed if the ſcoop commonly employed in ſuch caſes had been incautiouſly introduced.

