# Erratum

**Published:** 1786

**Authors:** 


E R R A T U M.

In page 37, lines 22 and 23 fhould be arranged in the following manner, viz.

Flofculi umbellæ feffilis fertiles.

—— ——- pedunculatæ plerumque abortiunt.

